# Pregnancy screening practices and treatment of pregnant patients among radiation oncologists: results of an international survey

**DOI:** 10.3332/ecancer.2021.1169

**Published:** 2021-01-13

**Authors:** Peter Zaki, Junjia Zhu, Heath B Mackley, Jennifer C Rosenberg

**Affiliations:** 1 Department of Radiation Oncology, Penn State Cancer Institute, 400 University Dr, Hershey, PA 17033, USA; 2 Department of Radiation Oncology, University of Washington, 1959 NE Pacific St, Seattle, WA 98195, USA; 3 Department of Public Health Sciences, Penn State Cancer Institute, 400 University Dr, Hershey, PA 17033, USA; a https://orcid.org/0000-0002-7451-4712

**Keywords:** survey, ASTRO, policy, foetus, embryo

## Abstract

**Background:**

The human embryo or foetus is susceptible to harmful effects of radiation, which include growth delay, malformations, impaired cognitive function, cancer and foetal demise. The purpose of this study is to describe pregnancy screening practices in radiation oncology, so that potential health effects may be avoided and areas of prevention may be identified.

**Methods:**

An electronic survey was delivered to 6,304 members of the American Society for Radiation Oncology. The survey subjects were radiation oncologists who are currently practicing in the world. Chi-square tests and a multiple logistic regression model were used to analyse the data. All tests were two-sided and the statistical significance level used was 0.05. This study (STUDY00009765) was approved by an Institutional Review Board.

**Results:**

A total of 434 responses from practicing radiation oncologists were received. Of these respondents, 69.1% were practicing in the United States. Of all respondents, 19.8% reported treating paediatric patients and 93.6% reported treating premenopausal patients. Despite 84.8% of radiation oncologists saying they would ‘strongly agree’ or ‘agree’ that one should screen for pregnancy prior to radiation therapy, 29.7% of respondents reported their department has no screening policy and 7.1% of respondents reported they do not screen for pregnancy. Having a departmental policy was associated with screening for pregnancy (*p*-value = 0.0005).

Of all respondents, 93 reported treating a known pregnant patient. Of these 93 respondents, 76 reported intentionally treating and 17 reported accidentally treating a pregnant patient. Respondents who did not screen at time of simulation were significantly more likely to treat a pregnant patient than those who screened at time of simulation (*p*-value = 0.0459).

**Conclusions:**

Heterogeneity exists among practicing radiation oncologists regarding pregnancy screening. Institutional policies should be clear and consistent. All members of the radiation oncology team should make every effort to minimise unintended radiation exposure to the embryo or foetus.

## Background

The pregnancy status of female patients presenting for radiation therapy is not always known. Unfortunately, patient history alone is not reliable for determining the possibility of pregnancy [1–8]. In 1975, Laubach and Wilchins recommended that a pregnancy test be performed on any female of childbearing age before diagnostic or therapeutic interventions to avoid potentially teratogenic complications and miscarriage [9, 10]. Patients might be screened prior to interventions (e.g., surgery or chemotherapy), but patients undergoing radiation therapy may begin treatment weeks after these therapies are completed. Furthermore, definitive or adjuvant radiation therapy may last several weeks. Compared to one-time interventions, this raises unique questions regarding optimal timing for pregnancy screening.

The harmful effects of radiation are directly related to the amount of radiation exposure [11]. The threshold dose for noncancer effects is dependent on the time of radiation post-conception [11–15]. For the first 16 weeks postconception, the radiation exposure threshold for noncancer effects is likely 0.05–0.30 Gy [10–14]. After 16 weeks postconception, the threshold is 0.30–0.50 Gy [10–14]. An embryo is most susceptible to major malformations during organogenesis (2–8 weeks postconception) [11]. The foetus is most susceptible to growth restriction and mental disability in its early phase (8–15 weeks postconception) [11]. In a cohort of patients exposed *in utero* to radiation from the atomic bombs in Japan, a linear dose–response model of their IQ showed a mean loss of about 25–31 points per 1 Gy beyond 0.1 Gy [16]. In the same cohort, patients who received doses greater than 1 Gy had a permanent 3%–4% reduction in height by the age of 18 years [17]. *In utero* exposure to any ionising radiation regardless of dose is associated with an increased risk of childhood malignancy, especially leukaemia [18, 19]. There is also evidence for elevated lifetime risk of cancer, particularly solid tumours [20]. For example, *in utero* exposure to a radiation dose of 0.05 Gy increases the estimated lifetime risk for malignancy by 2% [21].

Radiation is a known cause of foetal complications, yet there are no consensus guidelines on pregnancy screening prior to radiation therapy. In contrast, the American Society of Anesthesiologists [22] and American College of Radiology [23] have guidelines for pregnancy screening prior to surgery and imaging, respectively. In the realm of radiation oncology, it is also imperative to understand current practices on pregnancy screening, so that policies can be improved and potentially avoid unintentional harm to embryos and foetuses. This study reports the results of an international survey and assesses pregnancy screening practices by radiation oncologists.

## Methods

An electronic survey was delivered to 6,304 members of the American Society for Radiation Oncology (ASTRO) using a list compiled by leaders of ASTRO. While membership includes radiation oncologists, physicists, therapists, dosimetrists and nurses, inclusion criteria for survey subjects were radiation oncologists who are currently practicing in the world. There were no exclusion criteria. The survey consisted of 16 questions amalgamated by the authors. The survey was open for a total of 30 days, and three reminders were sent following the original email. Demographic questions collected information on age, gender, level of experience, practice location, practice type, practice size, patient volume and age groups of patient treated. A five-point Likert scale was used to assess radiation oncologists’ opinions on pregnancy screening. Pregnancy screening questions assessed factors that affect the decision to screen, method of screening, time of screening, departmental policy and treatment of pregnant patients. The survey was not tested or validated prior to distribution. The average amount of time to complete the survey was about 3 minutes. This study (STUDY00009765) was approved by an Institutional Review Board.

### Statistical methods

Descriptive statistics such as the frequency counts and relative frequency proportions were generated for all participants and then by groups. Chi-square tests were used to examine the associations between the groups and selected factors. Sub-group analyses were performed according to a certain selection criterion (e.g., all United States (US) participants). For US radiation oncologists, a multiple logistic regression model was set up to examine the screening preferences against selected predictors. The results were reported as odds ratios with their 95% confidence intervals. All tests were two-sided and the statistical significance level used was 0.05. All analyses were done using R programming language version 3.5.1 (R Foundations).

## Results

### Overall respondent characteristics and patterns

Demographic data and pregnancy screening practices of all respondents are summarised in Tables 1 and 2, respectively. A total of 434 responses were received (response rate 6.9%) from a total of 27 countries. There were 300 US respondents with 89 respondents from the northeast, 77 from the south, 68 from the midwest and 66 from the west. One hundred and thirty-four respondents reported practicing outside of the US, and although 65 did not specify which country, the most common were Canada (12), Australia (5), Netherlands (5), Brazil (4), Germany (4) and Greece (4). Of all 434 respondents, 31 (7.1%) reported they do not routinely screen for pregnancy. Of all respondents, 93 (21.4%) reported treating a known pregnant patient. Of these reported cases, 76 (81.7%) respondents reported intentionally treating (Table 3) and 17 (18.3%) respondents reported accidentally treating a pregnant patient. Of these 93 cases, 14 (14.9%) resulted in abortions (10, or 10.8% were intentional), and 13 (13.8%) resulted in healthy babies. The remaining 66 (71.0%) respondents reported insufficient outcome data on the baby. Of all respondents who reported treating a known pregnant patient, 28% had a change in treatment plan following the positive pregnancy test. Demographic data and pregnancy screening practices are further dichotomised by US and non-US respondents in Tables 1 and 2, respectively.

### US respondent characteristics and patterns

#### Pregnancy screening preferences

A multiple logistic regression model showed radiation oncologists who strongly agreed or agreed one should screen for pregnancy prior to radiation therapy were significantly more likely to have a departmental policy (OR 15.4, 95% CI 5.75–46.3; *p*-value < 0.0001). In the US, preference on pregnancy screening was not associated with age (OR 0.36, 95% CI 0.06–1.54; *p*-value = 0.1937), gender (OR 1.13, 95% CI 0.39–3.45; *p*-value = 0.8259), yearly volume of female patients (OR 0.37, 95% CI 0.13–1.05; *p*-value = 0.0659), level of experience (OR 1.95, 95% CI 0.65–6.04; *p*-value = 0.2347), practice size (OR 0.79, 95% CI 0.26–2.43; *p*-value = 0.6748), practice type (OR 1.40, 95% CI 0.65–3.19; *p*-value = 0.4005) and treating a pregnant patient (OR 0.25, 95% CI 0.09–0.69; *p*-value = 0.0075). For the 300 US respondents, the most common methods of screening were a universal urine pregnancy test (32.0%), asking the patient about last menstrual period (LMP) and ordering a urine test if > 1 month and premenopausal (21.3%), a universal serum pregnancy test (18.0%), and asking the patient about LMP and ordering a serum test if > 1 month and premenopausal (13.7%).

#### Treatment of pregnant patients

Respondents who did not screen at time of simulation were significantly more likely to treat a pregnant patient than those who screened at time of simulation (*p*-value = 0.0459). Respondents with more than 20 years of experience were more likely than those with less than 20 years of experience to report treating a pregnant patient (*p*-value = 0.0011). While only 11.1% of respondents in the northeast and 17.6% of respondents in the south treated pregnant patients, 28.6% of respondents in the west and 30.4% of respondents in the midwest reported treating a pregnant patient (*p*-value = 0.0084). Having treated a known pregnant patient was not associated with method of screening (*p*-value = 0.7688), not screening for pregnancy (*p*-value = 0.0566), screening at time of consult (*p*-value = 0.6542), repeating screening during treatment (*p*-value = 0.3332) and having a departmental policy (*p*-value = 0.3699).

#### Departmental policy

Regarding departmental practice in the US, groups consisting of three or more radiation oncologists were more likely to have a departmental policy (*p*-value = 0.0025). The region in the US (*p*-value = 0.1320) and practice type (*p*-value = 0.1596) were not significantly associated with having a departmental policy. Regarding departmental policy, 96.2% of respondents with a departmental policy, compared to 68.4% of respondents without a departmental policy, reported strongly agreeing or agreeing that one should screen for pregnancy prior to radiation therapy (*p*-value < 0.0001). Additionally, 8.8% of respondents without a departmental policy did not screen for pregnancy, while only 0.4% of respondents with a departmental policy reported not screening for pregnancy (*p*-value = 0.0005). Furthermore, a multiple logistic regression model was performed and found that strongly agreeing or agreeing that one should screen for pregnancy was associated with having a departmental policy (odds ratio 15.31; 95% confidence interval, 6.02–42.95).

## Discussion

There is a variation among radiation oncologists when it comes to screening for pregnancy. Patient age had the greatest impact on radiation oncologists when deciding to screen (Table 2). Most respondents (84.8%) strongly agreed or agreed that one should screen for pregnancy prior to radiation therapy. However, only 67.1% of respondents reported their department has a pregnancy screening policy (Table 2), which is a finding consistent with a 2016 survey conducted of National Comprehensive Cancer Network member institutions that found 30% of institutions did not have a pregnancy screening policy/recommendation prior to radiation therapy, chemotherapy or surgery [24]. Taken together, these findings suggest that the majority of radiation oncologists are in support of pregnancy screening and implementing departmental policies. The most common screening method was to ask a premenopausal patient about her LMP and order a urine test if it was greater than 1 month (25.6%) (Table 2). The urine test has both a sensitivity and specificity > 99%, while the serum test has a sensitivity of 97% and specificity of >99% [25]. The urine test may be more sensitive because of the need to maintain the lower limit for serum tests at 25 IU/L, instead of 20 IU/L, in order to avoid false positives from the destruction of the blastocyst [25]. The cost of a single urine test is $1.49 [26]. The urine pregnancy test may be the most popular choice for screening because it is both accurate and inexpensive [4, 27].

Respondents slightly more commonly preferred screening for pregnancy at time of simulation (53.7%) than at time of consultation (51.8%) (Table 2). In addition, respondents who did not screen for pregnancy at time of simulation were more likely to treat pregnant patients (*p*-value = 0.0459). The authors believe that pregnancy screening should be performed immediately before the simulation as it is closer to time of radiation delivery than time of consultation. Similarly, presurgical patients undergo pregnancy testing on the day of surgery [28]. Based on this study alone, it is unclear how often pregnancy screening should be repeated during treatment. Compared to one-time interventions (e.g. surgery) or diagnostic imaging, radiation therapy given over several weeks presents a unique challenge for pregnancy screening.

Nevertheless, pregnancy screening should be performed by radiation oncologists to avoid potential unintended negative consequences for the embryo or foetus. While 17 (3.9%) respondents reported accidentally treating a pregnant patient, there may be instances when the decision is made to treat a patient with known pregnancy as demonstrated by 76 (17.5%) respondents. Pregnant patients may elect to proceed with radiation therapy cautiously or may elect to have an abortion (see Table 3). Of the 93 (21.4%) respondents who reported intentionally or accidentally treating a known pregnant patient, 14 (14.9%) reported patients having abortions, which is likely an underestimate as 66 (71.0%) respondents did not provide additional data on the outcome of the foetus. Of all respondents who treated a known pregnant patient, 26 (28%) reported a change in treatment plan following the positive pregnancy test. This may be underestimated for two reasons. Firstly, respondents who reported treating a known pregnant patient may have either known the patient was pregnant before or after delivering radiation therapy. Secondly, the survey did not specifically ask if there was a change in treatment plan.

The data of this survey suggests that having a departmental policy is associated with respondents screening for pregnancy (*p*-value = 0.0005) and strongly agreeing or agreeing that one should screen for pregnancy prior to radiation therapy (OR 15.4, 95% CI 5.75–46.3; *p*-value < 0.0001). Our institution recently implemented a departmental screening policy. Indications for testing were female patients between menarche and menopause (as defined by 1-year without known menses). Exclusion criteria were females with history of hysterectomy/bilateral oophorectomy, tubal ligation, previous radiation therapy to the pelvis or known pregnancy. Patients with an indication for testing undergo a urine pregnancy test within 24 hours of simulation. If a patient declines testing, a waiver is signed by the patient or legal guardian, and the attending/designated physician is required to discuss the risks of proceeding without a test. Both the waiver and discussion are documented in the electronic health record. In the case of a negative test, the result is documented, and the simulation proceeds as scheduled. If the test is positive, the attending/designated physician is notified immediately to discuss the result with the patient.

To date, the prevalence of unrecognised pregnancy in radiation oncology patients is unknown. This may be because there is inconsistent pregnancy screening in patients receiving radiation therapy. In addition, the only study of pregnancy screening in radiation oncology patients at one institution did not report prevalence of pregnancy [29]. The study analysed 131 female patients between the age 12 and 55 years over the course of 1 year [30]. Patients determined to be ‘at risk’ or have a uterus were 95 (72%) [29]. Of all ‘at risk’ patients, testing was done in 45 (47%), but testing was performed within 14 days of starting radiation therapy for only 16 (17%) of cases [29]. In contrast to radiation oncology, several studies have analysed the prevalence of unrecognised pregnancy in surgical patients. The prevalence of unrecognised pregnancy in women with childbearing potential presenting for ambulatory, non-obstetric surgery was reported to be 0.3% [30]. In adolescent surgical patients, unrecognised pregnancy rates were reported to be 0.5% [31], 0.9% [4] and 1.2% [1]. For adult surgical patients, the prevalence was even higher at 2.2% [32]. The prevalence in radiation oncology may be roughly estimated by the following. The National Cancer Institute estimated about 1.8 million new cancer cases in the US in 2018 [33], and the International Agency for Research on Cancer estimated 18.1 million new cancer cases worldwide in 2018 [34]. Assuming that women make up half of the new cases of cancer each year and half of cancer patients will receive radiation therapy [35], about 450,000 female patients present for radiation therapy each year in the US. The American Association of Physicists in Medicine estimated 4,000 pregnant patients required radiation therapy in 1 year in the US [36]. This would mean the unrecognised pregnancy prevalence rate in radiation oncology patients is about 0.9%. The longer a radiation oncologist is in practice, the higher the probability that one will interface with a pregnant patient. In fact, the current study found that respondents with more than 20 years of experience were more likely to report treating a pregnant patient than those with less than 20 years of experience (*p*-value = 0.0011). This emphasises the importance of keeping pregnancy screening in mind throughout one’s practice.

Limitations of this study include its inability to determine the most effective pregnancy screening practice due to its retrospective nature. However, the results of this study suggest the optimal time of testing is at time of simulation. Respondents were susceptible to recall bias (e.g. those who treated a known pregnant patient and had negative outcomes may be more likely to remember, and thus report treating a pregnant patient). Survey respondents also constituted a small sample size of radiation oncologists and were susceptible to participation bias. For example, radiation oncologists who treated a known pregnant patient and had a negative outcome may either decide to fill out the survey to teach other practitioners from their experience or avoid filling out the survey, in partial or full, out of fear of possible repercussions. Although the response rate was 6.9% (434 respondents), previous response rates of surveys distributed using the ASTRO membership database ranged from 10.0% to 15.8% (574–649 respondents) [37–39]. The response rate in this study may be lower because the contact list was compiled in 2010 and some contacts were non-radiation oncologists; therefore, some emails were outdated and some members in the directory did not meet the inclusion criteria to partake in the survey. In calculating the response rate, these issues could not be accounted for, and thus resulted in an underestimate. Despite the small response rate, there was representation across the US and the world from both academic and private groups. Another limitation of this study is that some radiation oncologists may not directly screen for pregnancy, but rather auxiliary staff may screen. Although this may be the case, radiation oncologists are responsible for safe delivery of radiation and are usually aware of their patients’ pregnancy status as evidenced by the results of this study.

All patients are entitled to counselling before exposure to radiation [11, 40]. If a radiation oncologist does not investigate the possibility of pregnancy, he or she risks causing irreversible foetal damage, foetal demise or increased chance of paediatric cancer [41]. Additionally, radiation physicists should be consulted for special planning of pregnant patients if treatment is to proceed. This study lays the foundation for further investigation. Future research may focus on reasons why radiation oncologists do not screen and reasons why patients decline testing. Furthermore, an effective and generalisable screening method should be determined and implemented.

## Conclusion

This study describes current pregnancy screening practices by radiation oncologists in the world. There is no uniform pregnancy screening practice for patients receiving radiation therapy, and an effective algorithm is recommended to provide the best possible outcomes. The results of this survey indicate that having a departmental policy is associated with radiation oncologists screening for pregnancy. Our findings also highlight that screening at time of simulation is the most preferred time to screen and may be associated with less treatment of pregnant patients. In order to avoid unintended harm to the patient and embryo/foetus, we recommend routine screening for pregnancy in patients receiving radiation therapy. In cases of a positive test result, the patient should receive appropriate counselling, and radiation physicists should be consulted. Every department should establish a clear and consistent pregnancy screening policy based on law and ethics. Individually, all members of the radiation oncology team should make every effort to minimise unintended radiation exposure to the embryo or foetus.

## Conflict of interest

The authors declare that they have no conflict of interest.

## Funding statement

The authors have no funding to declare.

## Figures and Tables

**Table 1.** Demographic data of all respondents.

	Total respondents	US respondents	Non-US respondents	*p*-value
Number of respondents	434	300 (69.1%)	134 (30.9%)	
Age (years)				0.1248
<25	0 (0.0%)	0 (0%)	0 (0%)	
25–34	1 (0.2%)	1 (0.3%)	0 (0%)	
35–44	116 (26.7%)	71 (23.7%)	45 (33.6%)	
45–54	155 (35.7%)	106 (35.3%)	49 (36.6%)	
55–64	124 (28.6%)	95 (31.7%)	29 (21.6%)	
≥65	38 (8.8%)	27 (9.0%)	11 (8.2%)	
Gender				0.7898
Male	268 (61.8%)	187 (62.3%)	81 (60.4%)	
Female	166 (38.2%)	113 (37.7%)	53 (39.6%)	
Yearly volume of female patients				0.0537
≤50	39 (9.0%)	27 (9.0%)	12 (9.0%)	
51–-100	89 (20.5%)	61 (20.3%)	28 (20.9%)	
101–200	148 (34.1%)	103 (34.3%)	45 (33.6%)	
201–300	75 (17.3%)	61 (20.3%)	14 (10.4%)	
301–400	33 (7.6%)	22 (7.3%)	11 (8.2%)	
401–500	18 (4.2%)	10 (3.3%)	8 (6.0%)	
>500	32 (7.4%)	16 (5.3%)	16 (11.9%)	
Level of experience				0.1545
In residency	2 (0.5%)	0 (0.0%)	2 (1.5%)	
<5 years out of training	11 (2.5%)	9 (3.0%)	2 (1.5%)	
5–10 years out of training	72 (16.6%)	50 (16.7%)	22 (16.4%)	
11–20 years out of training	155 (35.7%)	100 (33.3%)	55 (41.0%)	
21–30 years out of training	130 (30.0%)	95 (31.7%)	35 (26.1%)	
>30 years out of training	64 (14.8%)	46 (15.3%)	18 (13.4%)	
Radiation oncologists in group				0.0009*
1–2	81 (18.7%)	69 (23.0%)	12 (9.0%)	
3–-5	130 (30.0%)	87 (29.0%)	43 (32.1%)	
6–10	93 (21.4%)	64 (21.3%)	29 (21.6%)	
11–15	55 (12.7%)	28 (9.3%)	27 (20.1%)	
>15	75 (17.3%)	52 (17.3%)	23 (17.2%)	
Practice type				<0.0001*
Private	207 (47.7%)	161 (53.7%)	46 (34.3%)	
Academic	172 (39.6%)	113 (37.7%)	59 (44.0%)	
Government	25 (5.8%)	5 (1.7%)	20 (14.9%)	
Other	30 (6.9%)	21 (7.0%)	9 (6.7%)	
Patient age groups				
Paediatric	86 (19.8%)	56 (18.7%)	30 (22.4%)	0.4424
Premenopausal adults	406 (93.6%)	284 (94.7%)	122 (91.0%)	0.2273
Postmenopausal adults	427 (98.4%)	298 (99.3%)	129 (96.3%)	0.0537

* US respondents were more likely to have smaller group sizes and more likely to have private groups than non-US respondents

**Table 2.** Pregnancy screening practice data of all respondents.

	Total respondents	US respondents	Non-US respondents	*p*-value
Screening preference				<0.0001*
Strongly agree	270 (62.2%)	209 (69.7%)	61 (45.5%)	
Agree	98 (22.6%)	64 (21.3%)	34 (25.4%)	
Neither agree or disagree	50 (11.5%)	22 (7.3%)	28 (20.9%)	
Disagree	14 (3.2%)	5 (1.7%)	9 (6.7%)	
Strongly disagree	2 (0.5%)	0 (0%)	2 (1.5%)	
Decision factors for screening				
Patient age	404 (93.1%)	289 (96.3%)	115 (85.8%)	0.0002*
History of hysterectomy/oophorectomy	270 (62.2%)	214 (71.3%)	56 (41.8%)	<0.0001*
Use of contraception	171 (39.4%)	121 (40.3%)	50 (37.3%)	0.6252
Body part receiving radiation	102 (23.5%)	57 (19%)	45 (33.6%)	0.0014*
History of prior pelvic radiation	81 (18.7%)	52 (17.3%)	29 (21.6%)	0.3519
Method of screening				<0.0001*
Ask patient about LMP and order urine test if >1 month and premenopausal	111 (25.6%)	64 (21.3%)	47 (35.1%)	
Universal urine pregnancy test	108 (24.9%)	96 (32.0%)	12 (9.0%)	
Universal serum pregnancy test	69 (15.9%)	54 (18.0%)	15 (11.2%)	
Ask patient about LMP and order serum test if >1 month and premenopausal	63 (14.5%)	41 (13.7%)	22 (16.4%)	
Other	51 (11.8%)	39 (13.0%)	12 (9.0%)	
I do not ask about LMP or test for pregnancy	13 (3.0%)	2 (0.7%)	11 (8.2%)	
I do not test for pregnancy but I ask the patient to do home test	7 (1.6%)	1 (0.3%)	6 (4.5%)	
I do not test for pregnancy but I ask the patient to do test at her OBGYN physician	6 (1.4%)	1 (0.3%)	5 (3.7%)	
I do not ask about LMP but will test for pregnancy if the patient requests	6 (1.4%)	2 (0.7%)	4 (3.0%)	
Time of screening				
Simulation	233 (53.7%)	205 (68.3%)	28(20.9%)	<0.0001*
Consultation	225 (51.8%)	133 (44.3%)	92(68.7%)	<0.0001*
Repeat during treatment	20 (4.6%)	14 (4.7%)	6(4.5%)	1
I do not screen for pregnancy	31 (7.1%)	7 (2.3%)	24 (17.9%)	<0.0001*
Departmental policy				<0.0001*
Yes	291 (67.1%)	237 (80.6%)	54 (42.9%)	
Treated known pregnant patient				0.8423
Yes	93 (21.4%)	63 (21.0%)	30 (22.4%)	

* US respondents were more likely to strongly agree one should screen for pregnancy and use universal screening tests than non-US respondents. US respondents were more likely to consider patient age and history of hysterectomy/oophorectomy and less likely to consider body part receiving radiation when deciding whether or not to screen for pregnancy. US respondents were more likely to screen at time of simulation whereas non-US respondents were more likely to screen at time of consultation. Non-US respondents were more likely to not screen for pregnancy. US respondents were more likely to have a departmental policy regarding pregnancy screening.

**Table 3.** Examples of reported deliberate treatment of pregnant patients.

Brain tumour patient in the third trimester. This was over a decade ago, and to date, no [known] difficulty with the child. We [checked] the dose via additional measurements including thermoluminescent dosimeters (TLDs) and added additional blocking.
Treatment to the shoulder during the third trimester. The position of the foetus was checked regularly with ultrasound. Dose predictions were checked using phantom measurements and clinically using diodes.
Spinal cord compression from small cell lung cancer in [a] 7-month pregnant woman who refused chemotherapy and refused termination. [The] child [was] ultimately delivered via C-section 4 weeks later. [The] mother died 2 months later.
Breast cancer [patient] became pregnant between chemo and beginning of RT; she refused abortion; we got genetics consultation, specially written informed consent, we took as much care as possible to shield lower pelvis and reduce scatter dose. She had a healthy baby, she sends us photos and her now-5-year-old’s artwork; no evidence of radiation ill effect.
During my residency, a patient in my department received RT for breast cancer and did not inform anyone that she was pregnant because she was afraid treatment for her cancer would be withheld. She ultimately delivered a healthy baby.
[Despite a] long [history] of amenorrhoea [due to] chemo, she conceived between [simulation] and start of definitive radiation therapy for cardiac angiosarcoma. [Pregnancy was] diagnosed ~3 [weeks] into course, [and she] had therapeutic termination 1 week later (I did not stop cardiac RT).
The patient was early with her pregnancy and had a neurotropic melanoma metastatic to her preauricular nodes. We treated her with electrons only with no image-guided radiation therapy. Lead apron over her [abdomen]. Dose to uterus and foetus was very low.
The patient was tested and [we] discussed not [to] get pregnant during treatment. She was not pregnant at the time of simulation. She started having nausea while getting breast radiation and was found to be pregnant. It was horrible experience for everyone. [The] patient eventually had a therapeutic abortion [of] her choice.
Patient was tested for pregnancy prior to simulation. She was counselled to use birth control during radiation therapy. She ended up getting pregnant during RT and she opted to have an abortion after RT (unrelated to concerns about foetal exposure; she did not wish to have more children).
We did [a] pregnancy test just before simulation, [and the] result [was] negative. Radiation treatment course commenced less than a month after CT–simulation, [which was] adjuvant whole breast in the prone position. We repeated pregnancy test 1 month [during the radiation therapy course] after the prior pregnancy test, and [the] result was [again] negative. The patient was having regular menses, however, was late. We repeated the pregnancy test before the usual 1-month interval given this information, and the result was positive for pregnancy. At that point, the course of adjuvant whole breast irradiation was nearly completed; however, tumour bed boost was pending. For this 36-year-old patient who has a significant other and who had just started trying to become pregnant just prior to her early stage node-negative breast cancer diagnosis, this was a surprise and was very emotional. We had full discussions with medical oncology, reproductive endocrinology and the patient and her partner. She wished to keep the pregnancy, and also continue and complete the radiation therapy course understanding the potential risks. Extensive physics measurements were performed which showed extremely low scattered dose to the gynaecologic organs. The patient completed the radiation therapy course without incident. She carried the pregnancy to term with close monitoring by reproductive endocrinology, and had an uneventful delivery of a healthy baby daughter.
